# Committees

**Published:** 1867-07

**Authors:** 


Dr. C. Goodbrake, in behalf of the Nominating Committee, made the following recommendations of committees:—

*For Local Secretary*—Dr. J. Robbins, of Quincy.

*For Committee of Arrangements*—Drs. Louis Watson, J. N. Ralston, and J. T. Wilson, of Quincy; M. Shepherd, of Payson; and W. M. Landon, of Burton.

*For Committee on Necrology*—Drs. J. H. Hollister, of Chicago; F. B. Haller, of Vandalia; and W. S. Edgar, of Jacksonville.

*For Committee on Legislation*—Drs. S. T. Trowbridge, of Decatur; H. A. Johnson, of Chicago; F. B. Haller, of Vandalia; W. S. Edgar, of Jacksonville; and H. Noble, of Heyworth.

*On Cholera-Infantum*—Dr. DeLaskie Miller, of Chicago.

On motion, the report was accepted, and its recommendations adopted.

Dr. H. A. Johnson offered the following, which was adopted:

*Resolved*, That the Committee on Legislation be instructed to prepare and present to the next regular session of the State Legislature, a bill legalizing human dissections, and that each member of this Society be requested to urge upon our legislators the importance of such legal provisions, as a protection to public and private cemeteries, as well as for the promotion of medical and surgical education.

Dr. D. W. Young moved that the thanks of the Society be tendered to Dr. J. Adams Allen, for his interesting public address last evening, and that he be requested to furnish a copy of the same to the Publication Committee, which motion was carried in the affirmative.

Dr. P. Bailhache, Chairman of the Committee on Drugs and Medicines, presented an interesting report, which was accepted and referred to the Committee of Publication.

Dr. David Prince read an abstract of his report on Plastic Surgery, which was accepted, and the report referred to the Committee of Publication.

Dr. H. A. Johnson offered the following, which was adopted:

*Resolved*, That Drs. J. W. Freer and Edmund Andrews be appointed by this Society, delegates to the International Congress, to be held in Paris, during the month of August next.

Dr. E. L. Holmes, Special Committee on Ophthalmology, presented a brief report, which was accepted and referred to the Committee of Publication.

Dr. T. D. Fitch, Chairman of the Committee on Specialties and Medical Advertising, presented a report, which was read by the Secretary and referred to the Committee of Publication.

Dr. D. Prince, from the same committee, presented a minority report, which was also referred to the Committee of Publication.

Dr. J. S. Hildreth read an interesting paper on Granular Conjunctivitis with Paniform Cornea, which was accepted and referred to the Committee of Publication.

Dr. H. A. Johnson presented some interesting illustrations of the pulse lines, as written by the sphygmograph, together with the instrument. The communication was referred to the Publication Committee.

Dr. N. S. Davis read a paper on the Physiological Effects of Alcoholic Drinks, which elicited an interesting discussion, and was referred to the Committee of Publication.

